# Long-Tailed Macaque (*Macaca fascicularis*) Contraception Methods: A Systematic Review

**DOI:** 10.3390/biology12060848

**Published:** 2023-06-13

**Authors:** Muhammed Mikail, Tengku Rinalfi Putra Tengku Azizan, Mohd Hezmee Mohd Noor, Hasliza Abu Hassim, Azlan Che’Amat, Mohd Qayyum Ab Latip

**Affiliations:** 1 Department of Veterinary Preclinical Sciences, Faculty of Veterinary Medicine, Universiti Putra Malaysia, Serdang 43400, Malaysia; ningimikail74@gmail.com (M.M.); hezmee@upm.edu.my (M.H.M.N.); haslizaabu@upm.edu.my (H.A.H.); qayyum2188@gmail.com (M.Q.A.L.); 2 Wildlife Research Centre, Faculty of Veterinary Medicine, Universiti Putra Malaysia, Serdang 43400, Malaysia; c_azlan@upm.edu.my; 3 Institute of Tropical Agriculture and Food Security (ITAFoS), Universiti Putra Malaysia, Serdang 43400, Malaysia; 4 Department Veterinary Clinical Studies, Faculty of Veterinary Medicine, Universiti Putra Malaysia, Serdang 43400, Malaysia

**Keywords:** reproduction, hormonal and non-hormonal contraception, sex steroid, hormone antagonist and fertility control

## Abstract

**Simple Summary:**

Long-tailed macaques have been able to withstand the pressure of human activities and adapt well to reproduce in an anthropogenic environment. These increased human–macaque interactions bring about direct and indirect effects on humans, notably, the risk of zoonotic disease transmission, nuisance and aggressive behaviors toward tourists, and losses to farmers due to crop raids; as a result, they are regarded as pest primates. Culling and other lethal control methods are a few population control methods employed to address the pest behavior effect of long-tailed macaques, but they prove ineffective. However, contraception methods involving hormonal and non-hormonal methods are reported to have variable success rates in the long-tailed macaque. Knowledge about contraception and its side effects on the wild macaque is inadequate; therefore, future studies are required to address these issues in order to prove that contraception methods are better alternative to lethal population control methods.

**Abstract:**

The contraception-based approach to wildlife management is a humane and effective alternative to population control methods. Wildlife management only has a few conventional ways to control overpopulation, such as culling, translocation, poisoning, and allowing natural death. Nevertheless, these methods usually have short-term, lethal, and unethical effects. The present systematic review aims to review the knowledge on contraception reported in long-tailed macaques as an alternative to population control. We obtained 719 records from searching CABI, PubMed, ScienceDirect, and Scopus electronic databases. After the screening and selection process, according to PRISMA guidelines, 19 articles that met the eligibility criteria were chosen. Of the 19 articles, 15 were studies on female long-tailed macaque contraception methods (six (6) hormonal and nine (9) non-hormonal). We analyzed four (4) selected articles on male Cynomolgus monkey contraception methods (two (2) hormonal and two (2) non-hormonal). One of the nine (9) articles on female long-tailed macaque contraception reports negative results. Furthermore, only two (2) studies used free-ranging long-tailed macaques as test subjects, while seventeen (17) tested on captive ones. The challenges of long-tailed macaque contraception identified in this review were the effectiveness of the contraceptive, the administration route, the economic feasibility, the distinction between captive and free-ranging Cynomolgus macaques, the choice of permanent or reversible contraception, the capability of contraceptive use for population control, and the lack of studies on the free-ranging long-tailed macaque. Notwithstanding the literature gap on long-tailed macaque contraception for population control, long-tailed macaque contraception exhibits potential as an alternative method to culling long-tailed macaque. Future research should address these obstacles to support the long-tailed macaque contraception as an alternative population control method.

## 1. Introduction

The long-tailed macaque, also known as the cynomolgus monkey (*Macaca fascicularis*), has the second most widespread geographical distribution among primates after the rhesus macaques (*Macaca mulatta*) [1]. Its population is distributed throughout the southern part of the southeast Asian mainland, including Myanmar, Thailand, Laos, Vietnam, Cambodia, Peninsular Malaysia, Singapore, Sundaland (the islands of Borneo, Sumatra, and Java, as well as adjacent islands), and beyond (the islands east of the Wallace Line, Philippines) [1,2,3,4].

Long-tailed macaques have a history of coexisting with humans in Malaysia and adapt well to reproduce in anthropogenic environments [5]. In fact, during the past two decades, the number of long-tailed macaques has increased dramatically due to incidental food subsidies from household food waste. Karuppannan et al. [6] estimated that there are 127,050 long-tailed macaques in Peninsular Malaysia, with a projected 133,403 if they grow by 5% yearly [6]. The Macaques are reported to harbor and transmit specific zoonotic pathogens. These disease includes malaria, tuberculosis, influenza A and B, Simian Foamy Virus (SFV), and Macacine Herpesvirus 1 (MaHV1/B virus) [7,8,9]. The direct effect of long-tailed macaque overpopulation is a lack of psychosocial well-being and discomfort for tourists as well as losses for farmers due to the cost of crop guarding [10,11,12]. 

Studies indicates that long-tailed macaque overpopulation management methods, such as culling, are controversial and ineffective due to the population’s continued growth [13]. The negative response of animal rights activists in Malaysia to news of culling operations indicates that culling is a very contentious fertility control method [14,15]. Furthermore, it is not acceptable from an ethical point of view regarding the animals’ welfare [16,17]. 

Contraception refers to preventing ovulation, fertilization, the implantation of an embryo, and the continuation of pregnancy through various artificial means [18]. Furthermore, contraception addresses the issue of resource scarcity without causing a proportionate growth in population size, enhances individual well-being by alleviating resource scarcity, and manages certain diseases through contraception [19,20]. Contraception methods reported in macaques consist of hormonal and non-hormonal methods [18]. Hormonal contraception is a term that refers to techniques of contraception that work by affecting the endocrine system through the use of synthetic steroids and synthetic steroid antagonists [21]. In contrast, non-hormonal contraception is a method that neither uses hormones nor hormone antagonists for contraception purposes [22].

This article aims to systematically review current knowledge of contraception used in long-tailed macaques as a possible alternative to population control. The contraceptive methods reviewed are the hormonal/steroid contraceptive methods and the non-hormonal/chemical agent contraceptive methods.

### 1.1. Methodology

We used the updated Preferred Reporting Items for Systematic Reviews and Meta-Analysis (PRISMA) guidelines 2020 for this review [23].

### 1.2. Information Sources

We searched four primary databases that are reliable and provide full text. They are CAB Direct, Scopus, ScienceDirect, and PubMed, respectively. The search keyword combinations of # Cynomolgus macaque contraception” and # Long-tailed macaque contraception” were used for each database with the “AND” configuration.

### 1.3. Eligibility and Rejection Criteria

We identified specific eligibility and rejection criteria. We chose studies examining hormonal or non-hormonal contraception in long-tailed macaques (Cynomolgus monkey). Studies are included if they met the following criteria: only research or short communication articles, with full-text availability and written in English. Furthermore, studies are included if they involve either male Cynomolgus macaque or female Cynomolgus macaque or both as subjects and test the contraceptive efficacy of the method used.

The rejection criteria are as follows: review articles, book chapters, encyclopedias, conference proceedings and studies on subjects other than cynomolgus macaques, notwithstanding studies that did not test the efficacy of contraceptive for contraception but instead tested for an alternative function other than contraception, and those without a full text written in another language other than English are rejected from the study.

### 1.4. Selection Process

The Mendeley desktop’s duplication tool was used to find and eliminate duplicate articles. The full texts of the different studies were evaluated, and those that did not meet the criteria were excluded. The title and abstract were also screened.

### 1.5. Method of Data Collection and Synthesis

Our objective is to evaluate and analyze existing research articles discussing or reporting the effects of contraceptives agents on cynomolgus macaques. 

## 2. Results

The following are the individual data extracted from the eligible studies or articles and include the sex of the Cynomolgus macaque, the method of contraceptive administration, the type of contraception tested, and the findings of the contraceptive studies. The extracted data were analyzed using Microsoft Office Excel 2016 after the articles were divided into two categories based on the contraception used: hormonal and non-hormonal contraception methods in female and male Cynomolgus macaques. Furthermore, the methodological quality of each article was evaluated using the Mixed Method Appraisal Tool (MMAT) and sensitivity analysis, as described in [24].

### 2.1. Study Selection

**Figure 1:**
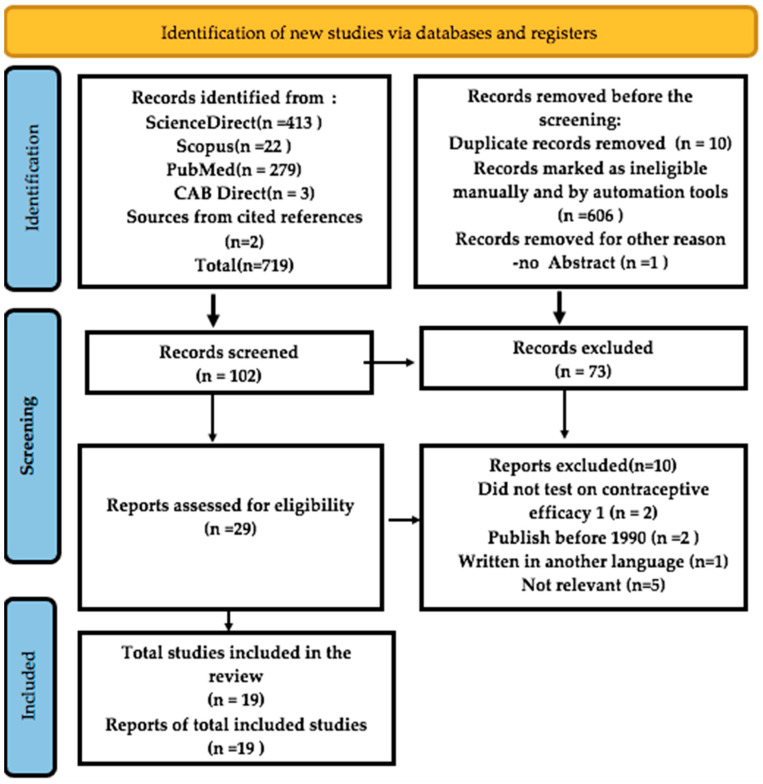
The Flowchart diagram of the study showing the selection process according to [23].

The information for this systematic review is drawn from four databases, as shown in Figure 1. Figure 1 shows that 719 records were found, screened, and carefully reviewed to be part of this review.

Duplicate and ineligible records were removed, and the titles and abstracts were checked against the criteria for inclusion and exclusion. These led to the removal of 617 documents. When the articles were checked for eligibility, 73 were taken out because they were review articles, book chapters, conference papers, or entries in an encyclopedia. Nineteen (19) of the remaining 29 articles passed the inclusion criteria screening and were thus included in this review. The study analyzed 19 articles.

### 2.2. Characteristics of the Included Articles

Out of the 19 included articles, eight (8) studies tested hormonal contraception, and eleven (11) tested non-hormonal contraception, as shown in Table 1 and Table 2. Furthermore, four (4) studies report contraception in male Cynomolgus macaques, while 15 studies report contraception in female Cynomolgus macaques. Except for the studies by [25,26], all the included studies report contraception in captive long-tailed macaques.

### 2.3. Hormonal Contraception

Studies involving hormonal contraception analyzed include progestin (2/8), androgen ester (1/8), GnRH antagonist (1/8), antiestrogen (1/8), and antiprogestin (3/8), respectively.

Hormonal contraception is a term that refers to techniques of contraception that work by affecting the endocrine system through the use of synthetic steroids and synthetic steroid antagonists [21]. Hormonal contraception acts directly on the central nervous system and reproductive organs by interfering with the hypothalamic–pituitary–gonadal axis [27,28] to inhibit ovulation or indirectly on the sex steroid receptors in the hypothalamus and anterior pituitary to inhibit gonadotropin release. Notwithstanding, inhibition of the release of gonadotropins causes a decrease in LH levels. A low level of LH causes the inhibition of LH-dependent synthesis of T by the ovarian theca cells [29], and these subsequently prevent the occurrence of ovulation [30] as well as a decrease in the level of endogenous sex hormones [28]. Furthermore, hormonal contraception acts on the reproductive organs by decreasing the passage of spermatozoa into the uterus through the alteration of the cervical mucus and decreasing the chances of implantation by altering the structure of the uterus [28,31].

For the studies involving treatment with progestin (levonorgestrel diet), Nayak et al. [32] investigated the effect of levonorgestrel on female long-tailed macaques at different doses, different phases of the menstrual cycle, and different times of ovulation. In this study, levonorgestrel was mixed with a vehicle (corn oil) and added to the food of 22 female macaques (*n* = 22). The macaque was fed a levonorgestrel diet for two weeks, and the results of the study indicate that ovulation occurs more than 12 days after the last daily administration of the levonorgestrel diet at a dose of 50 μg, as such daily administration of diet containing levonorgestrel at a dose of 50 μg for two weeks delayed ovulation.

The effects of medroxyprogesterone acetate (MPA) on menstrual cycles and its possible use as a contraceptive in macaques was investigated by Shimuzu et al. [33]. Thirty-nine female macaques (*n* = 39) are included in the study. The study found that a single subcutaneous injection of medroxyprogesterone acetate has the potential to control fertility by suppressing ovulation in a way that is reversible and depends on the dose [33].

Collins and Hodgen [34] investigated the role of midcycle preovulatory progesterone secretion by administering antiprogestin RU 486 to eighteen (18) sexually mature, normally ovulating long-tailed macaques on days 10–12 of their menstrual cycle. The study’s findings revealed that antiprogestin RU 486 increased the length of menstrual cycles, delayed the ensuing folliculogenesis, and effectively prevented the impeding midcycle gonadotropin surge [34].

Tarantal et al. [35] reports the role of estrogen in the implantation process and tamoxifen’s potential as a contraceptive. Sixty adult female Cynomolgus macaques were divided into a treatment group (*n* = 30) and a control group (*n* = 30). On day four after ovulation, a single oral dose of 5 mg/kg was administered through nasogastric intubation, while the control group only received methylcellulose/distilled water. The study shows that tamoxifen does not affect the long-tailed macaque’s conception rate or luteal phase. The study explained further that the failure of tamoxifen to exhibit contraceptive potential might be due to its rapid excretory or metabolic rate, as it is not detected in the serum or urine [35].

Mehta and Chatterton [36] investigated the anovulatory effect of Anordrin, a synthetic steroidal antiprogestin administered intramuscularly in sesame oil at variable doses. Mehta and Chatterton [36] discovered that a lower dose administered during the macaque menstrual cycle’s first three (3) days inhibited follicular development, which was manifested by delayed ovulation time. Furthermore, the findings of [36] contradict other findings, which report that women taking Anordrin as a postcoital contraceptive had anovulatory cycles [37]. However, this could be because they took the drug as early as their menstrual cycles, suggesting the reason for ovulation failure [36].

Heikinheimo et al. [38] investigated the antiprogestin RU486’s endocrine mechanism and site of action, which inhibit ovulation in the Cynomolgus monkey. In order to have a clear understanding of how RU 486 is reported to inhibit ovulation in women [39,40], the study involves six regularly cycling female Cynomolgus macaques in three menstrual cycles. Between days 2 and 22, the treatment cycle received daily intramuscular administration of 1 mg/kg of RU-486 in ethanol, while the control cycle received only an ethanol injection. The result of the study reveals that ovulation was inhibited in five out of six Cynomolgus monkeys. Furthermore, it showed that interference with hypothalamic processing and positive feedback signals is the primary mechanism of ovulation inhibition in the hypothalamus. In addition, the study could not distinguish whether RU 486 acted as an antiprogesterone or antiestrogen.

For the GnRH antagonist study, Bremner et al. [41] examined the effect of sperm production and serum levels of testosterone on the daily intramuscular administration of the gonadotropin-releasing hormone GnRH antagonist (Deterelix) along with the simultaneous replacement of testosterone. Synthetic analogs such as Deterelix are known to compete with GnRH for the pituitary binding site. These lower LH, FSH T, and inhibin levels in the blood to inhibit LH and FSH secretion. Male adult long-tailed macaques (*n* = 22) were divided into four distinct groups (*n* = 5) and given different doses of GnRH antagonists (250 μg/kg-day; 750 μg/kg-day) in addition to either a Sham implant for 12 weeks, a T via implant for 20 weeks and 16 weeks, or a vehicle alone for 20 weeks. Every two weeks, blood and seminal fluid samples were taken and analyzed. The study shows that combining a GnRH antagonist with T can successfully induce azoospermia. This suggests its effectiveness as a male contraceptive agent in the male Cynomolgus macaque. It effectively suppressed the serum levels of T. This suggests it could also treat androgen-dependent neoplasia.

The suppressive effect of testosterone buciclate, an androgen ester, in the male long-tailed macaque was investigated by Weinbauer et al. [42]. The study involved fifteen adult male macaques (*n* = 15) divided into three (3) groups with various treatments. The study found that testosterone buciclate diminishes serum LH’s bioactivity, suppresses FSH and inhibin serum levels, and reverses suppressive effects. Accordingly, the study concludes that inhibiting FSH rather than testicular androgen is required for spermatogenesis suppression in the male macaque.

### 2.4. Non-Hormonal Contraception

Non-hormonal contraception is a contraception method which neither uses hormone nor hormone antagonist for contraception purposes [22]. Articles on non-hormonal contraception included in these studies are herbs (2/11), phosphodiesterase (PDE) inhibitor (1/11), prostaglandin E2 receptor (1/11), non-steroidal anti-inflammatory drugs (NSAID) (2/11), surgical (2/11), chemical (2/11), and immunocontraception (1/11), respectively.

### 2.5. Male Non-Hormonal Contraception

In male non-hormonal contraception, two studies (2/11) were analyzed, involving herbs (1/2) and chemicals (1/2), respectively.

Chang et al. [43] attempted to comprehend the mechanism and specific site of action of triptonide in a proof-of-concept efficacy study on male Cynomolgus macaques. Triptonide, shown to exhibit a male antifertility effect [44], is a compound derived from the purification of a Chinese herb Tripterygium wilfordii F, which is used to treat a variety of autoimmune and inflammatory diseases in traditional Chinese medicine [45,46]. The study indicates that the oral administration of triptonide at a dose rate of 1.0 mg/kg BW for 5–6 weeks results in infertility in male macaques due to oligo-asthenoteratozoospermia, which ceases following the discontinuation of the drug administration. Furthermore, the triptonide disrupts its interaction with SPEM 1 during spermatogenesis at the junction of plakoglobin/gamma-catenin; hence, the best strategy for developing male contraceptives is to target spermatogenesis at the late stage, as shown by the results.

O’Rand et al. [47] investigated the effect of an organic compound, EP055, in semen on male Cynomolgus sperm motility. The studies also determined the compound’s plasma half-life after a single intravenous infusion for binding to its target site. According to the findings in Cynomolgus macaque, a single intravenous infusion of 63.25 mg/kg causes its detection in the testes and epididymis within 2–6 h after the infusion. The studies also used male rhesus macaques as trial animals to measure the compound’s efficacy. EP055 was found to have the potential to be a reversible male contraceptive in both macaque species.

### 2.6. Female Non-Hormonal Contraception

In female non-hormonal contraception, nine (9) studies (9/11) were analyzed involving herbs (1/9), phosphodiesterase (PDE) inhibitors (1/9), prostaglandin E2 receptors (1/9), non-steroidal anti-inflammatory drugs (NSAIDs) (2/9), surgical (2/9), chemical (1/9), and immunocontraception (1/9), respectively.

Deleuze et al. [26] performed a tubectomy on 140 reproductive long-tailed macaques (129 adults and 11 subadults) in the Ubud Monkey Forest in Indonesia. The procedure reports success in 97.1% of the tubectomized animals. These endoscopic tubectomy was found to be safe and effective with 100% reliability after three years of post-operative monitoring, with no new births recorded.

In a related study, Martelli et al. [25] performed laparoscopic tubectomies on 1343 cynomolgus and female rhesus macaques in Hong Kong over ten years (2009–2019). Tubectomies with ovarian conservation are recommended as a permanent sterilization option for wild macaques and other wild primates since this population control method results in infertility without the adverse effects of surgical menopause.

Hester et al. [48] investigated the effect of the COX-2 inhibitor meloxicam on preventing oocyte release without disrupting the normal menstrual cycle during the follicular phase of the menstrual cycle in the Cynomolgus macaque. The idea was to investigate the effect of COX-2 inhibitors because COX-2 activity is required for successful ovulation. As such, it was reported that drugs inhibiting COX-2 activity are effective as contraceptives, as evidenced by infertility observed in women taking such drugs [49].

The findings of Hester et al. [48] confirm and reveal for the first time that the oral administration of the COX-2 inhibitor meloxicam blocks the oocytes within the luteinized follicles. These findings are consistent with similar results of failed or delayed follicular rupture in women [50,51,52]. However, Jesan et al. [53] concludes that a five-day oral administration of meloxicam at a dose rate of 30 mg (the equivalent human dose) around ovulation time reduces the rate at which oocytes are released with no effect on the serum levels of estradiol and progesterone, and hence, it can be used as an emergency contraceptive in women.

Jesan et al. [53] investigated the potential of chronic administration of the phosphodiesterase (PDE) 3 inhibitor ORG 9935 to a female cycling macaque cohabiting with a male in a breeding group as a contraceptive in primates. Phosphodiesterase (PDE) 3 inhibitor ORG 9935, a derivative of carboxyimidamide, has been shown to inhibit oocyte maturation without affecting ovulation and selectively block the in vitro spontaneous resumption of oocytes in both rhesus and human oocytes. During controlled ovarian stimulation, when given to macaques, it does not affect the development of the corpus luteum of multiple follicles [54,55]. Sixteen adult female cycling macaques (*n* = 16) were used in the study and divided into equal treatment and control groups. The treatment group received 150 mg/kg of 9935 daily as an oral bait or once a week as an injection subcutaneously in Captex oil for six ovulatory cycles. After one ovulatory cycle, male animals were introduced to the group. The findings suggest that it prevents pregnancy when the PDE-3 inhibitor ORG 9935 is administered to a macaque at a serum concentration greater than 300 nmol/L. The effect is reversible once the treatment is stopped.

McCann et al. [56] also investigated the effect of oral administration of cyclooxygenase-2 inhibitors on reproductive function and their efficacy in preventing pregnancy in primates. McCann et al. [56] used an oral bait containing a dose of 0.5 mg/kg cyclooxygenase-2, using fruit as a vehicle. In contrast to Hester et al. [48], this study is divided into two categories: emergency contraception and the monthly contraception model. A total of 11 female Cynomolgus macaques (*n* = 11) were used in the emergency contraception model. The macaques were bred with a fertile male before ovulation and received oral meloxicam once a day for up to 5 days, beginning on the day of breeding. Three of the meloxicam treatment cycles resulted in pregnancy, representing a 6.5% pregnancy rate in the emergency contraception model treated with meloxicam compared to the same animals treated with oral vehicle only without meloxicam, where 5 out of 15 cycles yielded pregnancy, which represents 33.3%. These results concluded that the oral administration of meloxicam has an efficacy rate of 80% as an emergency contraceptive. In a monthly contraception model, female Cynomolgus macaques were divided into three treatment groups and cohabited with a male macaque for five days. The duration of treatment for each group varies. On cycle days 5 through 22, Group 1 (*n* = 9) received oral meloxicam, resulting in a 100% pregnancy. A pregnancy rate of 75% was achieved in the second group (*n* = 4) when oral meloxicam was taken every other day. The third group (*n* = 6) that spent five days caged with a male macaque and only received oral meloxicam for five days of breeding achieved an 83.3% pregnancy rate. As such, these studies conclude that meloxicam did not protect against pregnancy when female animals were allowed to cohabit freely. It is important to note that both studies use meloxicam and differ only in the experimental design. The study concludes that the pharmacological inhibition of COX-2 has potential for pregnancy prevention. As such, meloxicam could be an alternative method of emergency contraception. It is inexpensive, readily available, and has a serum half-life of approximately 20 h, making it suitable for once-daily use [49,50].

Peluffo et al. [57] investigates how PTGER2, a prostaglandin (PG) E2 receptor antagonist, reduces pregnancy in monkeys by blocking the periovulatory activities in the follicle without affecting the menstrual cycle. In these studies, 19 female Cynomolgus macaques (*n* = 19) were divided into two groups: the treatment group (*n* = 9) and the control group (*n* = 10). The treatment group received 10 mg/kg of PTGER2 subcutaneously twice daily for five months. On the other hand, the control group received 0.5 mL of sesame oil every day. Each group received a male macaque, who remained for the contraceptive trial. In contrast to the control group, which had 21 matings and 8 pregnancies out of 10 females, the study found that the treatment group had 2 pregnancies out of 9, despite a proven fertile male presence.

Despite the study’s finding that the administration of a non-hormonal PTGER2 reduces pregnancy rate, the two observed pregnancies might have resulted from the chronic drug treatment, which increased the metabolic pathway and caused the compound to degrade over time. Other possible local factors include redundancy in the action that controls the periovulatory event in the primate ovary. The studies concluded that treatment with PTGER2 antagonists reduces the rate of pregnancies without significantly affecting the menstrual cycle or the pattern of steroid hormones.

For the study involving herbs, Trisomboon et al. [58] investigated the effect of Pueraria mirifica (PM) on the menstrual cycle length and other related hormones. Pueraria mirifica, a typical traditional herb in Thailand, is reported to have phytoestrogens with numerous estrogenic potencies [59]. Based on the idea that the phytoestrogen isoflavones in Pueraria mirifica affect gonadotropins and ovarian hormone secretion by binding to the estrogen receptor in the pituitary or ovary, this study shows that the decrease in FSH and LH disrupts the menstrual cycle and inhibits ovulation [58]. It is essential to note that the present study could not eliminate the role of other phytoestrogens in the plant, such as miroestrol; as a result, this is a setback and highlights the importance of comprehending the effects of each phytoestrogen in the plant. 

Tollner et al. [60] investigated the contraceptive effect of lignosulfonic acid, a by-product of the sulfite-pulping process used to convert wood pulp into paper. The primate gametes were tested for lignosulfonic acid, and it was found to prevent the macaque sperm from binding to the zona pellucida and remain exposed on the surface of non-capacitated sperm. This finding contradict the results of other studies about the sperm receptors for the zona ligands that they remain unexposed until after capacitation [61,62]. Furthermore, it was discovered to be non-toxic and have an antifertility effect, making it suitable for vaginal contraceptives.

Gulyas et al. [63] investigated the antifertility effect of zona antibodies by immunizing twelve (12) long-tailed macaques with porcine zona pellucida and evaluated the serum antibody titer using radioimmunoassay. The findings of Gulyas et al. [63] indicate that six (6) out of twelve (12) monkeys conceived after weeks 6–10 of vaccination when their antibody titers were at the highest level. However, the remaining six have a lower antibody titer and were only conceived eight months after vaccination, when the antibody level markedly decreased. Gulyas et al. [63] conclude that porcine zona pellucida immunization has a reversible antifertility effect on the long-tailed macaque.

**Table 1:** Characteristics of the articles included that tested hormonal contraception.

Type of Contraception and Reference	Dose, Route of Administration and Duration	Mechanism of Action	Sample Composition	Studies Outcome/Efficacy
Female hormonal contraception				
[64] Levonorgestrel diet	(i) Pellet containing 0, 8, 20, 50 or 125 μg LNG daily from day 2 to 15 of the menstrual cycle (ii) 50 μg LNG Infusion into feeding pellet and orally administered daily on days 9–22, 16–29 or 23–36 of the menstrual cycle	It causes the elevation of FSH and LH and decreases E_2_ to inhibit ovulation in cynomolgus macaque by affecting the folliculogenesis and hypothalamus	Total sample size *n* = 22 (i) *n* = 4 in each treatment group, and control (ii) *n* = 4 each treatment days	(1). 8/22 (36.3%) treated @ 20 and 50 μg LNG experienced delayed ovulation (2). 4/22 (18.18%) treated @ 125 μg LNG experienced inhibited ovulation. (3). 4/22 (18.18%) treated @ 8 μg LNG failed to inhibit ovulation. (4). 4/22 (18.18%) control had normal ovulation. (5). 16/16 (100%) treated @ 50 μg LNG daily experienced delayed ovulation in all groups.
[33] Progesterone (Medroxyprogesterone acetate (MPA))	Single subcutaneous injection @ 15 mg/kg	Exerts its mechanism of contraception through the inhibition of follicular maturation and or inhibition of ovulation	Total sample *n* = 8 Treatment group *n* = 5 Control group *n* = 3	(1). 3/8 (37.5%) of control had no changes in the endocrine and menstruation profile. (2). 5/8 (62.5%) of treated showed complete cessation of menstrual cycles.
[34] Antiprogestin (RU 486)	Antiprogestin 5 mg/day, dexamethasone I mg/day and 0.5 mL ethanol, SID from cycle day 10 to 12	(61) indicates that RU 486 exerts its effect by acting as a progesterone antagonist, thereby affecting the physiologic pre-ovulatory gonadotropin surges	Total sample size *n* = 18 Treatment group (1) *n* = 5 Treatment group (2) *n* = 3 Treatment group (3) *n* = 3 Control group *n* = 5	(1). 5/18 (27.7%) of treated group 1 and 3/18 (16.6%) of treated group 2 showed a delayed rise in gonadotropin, delayed folliculogenesis and extended intermenstrual interval. While 3/18 (16.6%) of treated group 3 and 5/18 (27.2%) of control had a normal midcycle surge of gonadotropins.
[35] Antiestrogen (Tamoxifen)	A single oral dose of 5 mg/kg on day four post-ovulation	It exerts its antiestrogenic effect by competing with the endogenous estrogen for the estrogen–receptor complex	Total sample size *n* = 26 Treatment group *n* = 13 Control group *n* = 13	(1). In 6/13 (46%) of the treatment group, the cynomolgus monkey became pregnant while (2). 4/13 (31%) of the control group became pregnant
[36] Antiprogestin (Anordrin)	(i) 4.0 and 8.0 mg/kg b.w IM on days 9–13 of the menstrual cycle 0.1 or 0.2 mg/kg b.w IM within the first three days of the menstrual cycle	It exerts its effect through the inhibition of follicular development and subsequently inhibits ovulation	Total sample size *n* = 32 Control *n* = 8 (1) Treatment *n* = 8 (2) Treatment *n* = 7 (3) Treatment *n* = 3 (4) Treatment *n* = 5	(1). 8/32 (25%) of control values observed within the normal range. (2). 8/32 (25%) of treatment 1 and 7/32 (21.8%) of treatment 2 did not inhibit luteal activity and rather delayed the development of ovarian follicles. (3). 3/32 (9.3%) of treatment 3 observed the absence of menstruation for 5–6 months. (4). 5/32 (15.6%) of treatment 4 showed delayed follicular maturation with an increase in the length of luteal phase.
[38] Antiprogestin RU 486	1 mg/kg/day of RU486 IM for three consecutive cycles	RU 486 inhibits ovulation in cynomolgus macaque at the level of hypothalamic–pituitary axis by interfering with the feedback of ovarian periovolatory signals	*n* = 6 in 3 menstrual cycle (treatment, rest and control)	(1). In 6/6 (100%) of the treatment cycle, ovulation was inhibited during RU486 administration. (2). In 5/6 (83.3%) progesterone was undetected, while 1/6 (16.6%) had increased progesterone.
Male hormonal contraception				
[41] GnRH antagonist plus testosterone (Deterelix)	Silastic capsule implant (crystalline T) SC 5 days before injecting the GnRH antagonist GnRH antagonist injection @ 250 μg/kg, 750 μg/kg day	Exerts its action on cynomolgus monkeys through the reversible suppression of serum T, LH, FSH and inhibin levels and subsequently causes azoospermia due to combination of testosterone (Deterelix)	Total sample size *n* = 22 1. Treatment 1 *n* = 5 @ 250 μg/kg + Sham Implant 2. Treatment 2 *n* = 5@ 250 μg/kg+ T 3. Treatment 3 *n* = 5@ 750 μg/kg + T 4. Control *n* = 7	In animals treated with an antagoninst, 14/15 representing 93% became azoospermic including 9/10 of treated with antagonist plus T representing 90%. No changes observed in the control group.
[42] Testosterone bucilate (TB)	Control received diluent Treatment group 1 received TB@ 10 mg/kg Treatment group 2 received TB @ 20 mg/kg IM	Testicular involution is achieved through the complete suppression of FSH secretion	Total sample size *n* = 15 Control group *n* = 5 Treatment 1 group *n* = 5 Treatment 2 group *n* = 5	1. 5/15 (33.3%) of control had average LH levels and serum testosterone was within the normal range. 2. In 10/15 (66.6%) of both treated groups, the LH level dropped while 5/15 (33.3%) of the treated had a rise in serum testosterone level up to 4-fold and 5/15 (33.3%) of the treated had up to a 5-fold rise in serum testosterone.

**Table 2:** Characteristics of the articles included that tested on non-hormonal contraception.

Type of Contraception and Reference	Dose, Route of Administration, Duration	Mechanism of Action	Sample Size	Studies Outcomes
Male non-hormonal contraception				
[43] Triptonide (Tripterygium wilfordii)	0.1 mg/kg BW single oral dose	Exert its mechanism of action at its target site, the plakoglobin junction, during spermiogenesis disrupt its interaction with SPEM1. It causes deformed sperm, with compromised forward motility and low sperm count after 5 week of treatment	Total sample size *n* = 4 Control *n* = 2 + 2 female Treatment *n* = 2 + 2 female Each group was housed with 2 proven fertile females	2/4 (50%) of the treated had no pregnancy. 2/4 (50%) of the control had two pregnancies.
[47] EP055	IV infusion of a single dose of 63.25 mg/kg	It exerts its effect by targeting the sperm protein EPPIN on the surface of the spermatozoa to inhibit sperm motility	Total sample size *n* = 5 Infusion (IV)Treatment *n* = 3 Tissue level *n* = 2	3/5 (60%) of the infusion had 10.6 min half-life for EP055. 2/5 (40%) of the tissue demonstrated EP055 in the testis and epididymis 2–6 h after infusion.
Female non-hormonal contraception				
[26] Surgical approach	NA	Through laparoscopy. To carry out tubectomy	*n* = 140	No new pregnancy was recorded among the sterilized females, with a 96.3% survival rate.
[48] NSAIDs (Meloxicam)	Oral administration of meloxicam @ 0.5 mg/kg body weight/day in food for five days at mid-follicular, late follicular and periovulatory phases	Meloxicam in this study is thought to exert its contraceptive action by inhibiting the activity of ovarian prostaglandin, which affects ovulation by preventing follicular rupture and prevents the release of oocytes	Total sample size *n* = 24 Cycle 1(vehicle treatment) *n* = 4 × 3 group Cycle 2 (meloxicam treatment) *n* = 4 × 3 group Cycle 3 (vehicle oocyte) *n* = 4 × 3 group Cycle 4 (meloxicam oocytes) *n* = 3, *n* = 4 and *n* = 3	In both cycles 1 and 2, no significant differences were observed. In cycle 3, no oocytes in the luteinized follicles were observed. In cycle 4 8/11, 72.7% of oocytes were observed in the luteinized follicles.
[53] Phosphodiesterase (PDE) 3 inhibitor ORG 9935	Given orally @ 150 mg/kg SID or depot injection @ 150 mg/kg weekly for seven ovulatory cycles	ORG 9935 exerts its reversible contraceptive effect at a serum concentration above 300 nmol/L by inhibiting meiotic maturation of the oocyte without affecting ovulation or the normal function of the corpus luteum	Total sample size *n* = 16 Control *n* = 8 Treatment *n* = 8	In the control group, 7/8 become pregnant, representing 88% of the pregnancy rate. In the treatment group, 4/8 become pregnant, representing 50% of the pregnancy rate.
[25] Surgical approach	NA	Via laparoscopy to carry out tubectomy	N = 1343 comprising both cynomolgus and rhesus macaque	During the monitoring period from 2009 to 2019, none of the sterilized animals became pregnant, which was evidenced by the decrease in the population growth from under 60% to 30%.
[56] NSAID (Meloxicam)	Oral administration @ 0.5 mg/kg SID for five days after mating	Through the inhibition of prostaglandin synthesis, which interrupts the release of oocyte necessary for ovulation	Total sample size for emergency model *n* = 11 46 meloxicam treatment cycle 15 control cycle	In the oral vehicle, no meloxicam, the pregnancy rate was 5/15 (33%), while in meloxicam treatment group, the pregnancy rate was 3/46 (6.6%).
[57] Prostaglandin E2 receptor antagonist (PTGER2)/BAY06	Subcutaneously @ 10 mg/kg in 0.5 mL castor oil given BID for six months	BAY06 is thought to exert its effect through the inhibition of cumulus–oocyte expansion	Total sample size *n* = 19 Treatment group *n* = 9 Control *n* = 10	In the control, the pregnancy rate was 8/10, representing 80% pregnancy, while in the treated group, the pregnancy rate was 2/9, representing 22% pregnancy.
[58] Pueraria Mirifica (A Thai herb)	Suspension @ 10, 100, and 1000 mg/5 mL of distilled water/individual/day in 3 treatment group of macaque	The mechanism of its action involves the mediation of the estrogen receptor at the hypothalamic–pituitary–gonadal axis and the uterus, which reduces FSH and LH levels in the hypothalamus through a negative feedback mechanism. These factors subsequently influence the menstrual cycle and inhibit ovulation	Total sample size *n* = 9 Treatment group 1 @ 10 mg/5 mL *n* = 3 Treatment group 2 @ 100 mg/5 mL *n* = 3 Treatment group 3 @ 1000 mg/5 mL *n* = 3	1. 6/9 (66.6%) of treatment groups 1 and 2 had extended menstrual cycle length with recovery of hormones levels post-treatment. However, group 2 showed suppression of FSH and LH. 2. 3/9 (33.3%) of treatment group 3 showed complete cessation of the menstrual cycle and suppression of FSH and LH.
[60] Lignosulfonic acid	Involve treatment of sperm with 1.5 mg/mL before washing or after capacitation to evaluate sperm–oocyte interaction with a zona pellucida binding assay and invitro fertilization	It exerts its potential as a vaginal contraceptive by binding to the head of capacitated sperm, thereby inhibiting the binding of sperm to the zona pellucida	NA	92.5% of treatment with LSA inhibited sperm zona binding post-capacitation, while 82.5% inhibited sperm zona binding before washing. For in vitro fertilization, 19/19 (100%) of pre and post-capacitation treatment of sperm with LSA prevented fertilization following on the 19 oocytes, while 11/18 (61.1%) of control oocytes were fertilized
[63] Immunocontraception (Porcine Zona Pellucida)	360 μg IM on cycle day 26, followed by a booster of 180 μg IM at week two after the first two doses and four weeks apart from the rest	Immunocontraception with porcine zona pellucida in cynomolgus monkeys exerts contraception only when there is a sustained serum antibody level for several weeks	Total sample size *n* = 12 All animals were immunized with porcine zona pellucida	6/12 (50%) of immunized animals conceived, while 6/12 (50%) did not conceive.

## 3. Discussion

There have been studies of contraception methods that effectively prevent ovulation, fertilization, implantation, and the success of pregnancy [65]. Still, the following established criteria for ideal contraceptives that should be taken into account for free-roaming wildlife contraception are high efficacy, minimal or no side effects on animal physiology or welfare, agent flexibility and reversibility, without affecting the animal’s ability to reproduce, with no effect on the animal’s social structure, agent affordability, and remote delivery with a single administration [66].

These studies on the effectiveness of hormonal contraceptives in both male and female cynomolgus macaques (8/19 studies) showed that the hormonal method has much potential as an effective contraceptive that prevents ovulation and follicular maturation. Meanwhile, non-hormonal methods such as using chemicals (2/19 studies), non-steroidal anti-inflammatory drugs (NSAIDs) (2/19 studies), prostaglandin E2 receptor (1/19 studies), herbs (2/19 studies), immunocontraception (1/19 studies), and surgery (2/19 studies) had variable success rates in contraception in both male and female cynomolgus macaques.

Based on the results of this meta-analysis, most of the studies on hormonal contraceptives for cynomolgus macaques (8/19 studies) had promising results. Nevertheless, the non-hormonal studies (11/19 studies) require frequent ingestion or the precise timing of administration in relation to the breeding cycle. This means that its effectiveness may change with the environmental condition, which could hinder its successful implementation in free-ranging wildlife [67], affect the welfare of the treated animal due to frequent administration, and incur additional financial costs [68,69].

It is important to note here that all the hormonal contraception reported in these studies (8/19) exerts its mechanism of action through the hypothalamic gonadal axis (HPG axis) [27,28,29,30,31]. Notwithstanding the non-hormonal contraception methods in 2/11 studies, the gonads (testes) are their primary site for the mechanism of action [43,47], and 2/11 studies act through the gonads (ovary) to exert their contraception effect [48,53,56,57]. In addition, 2/11 studies achieved contraception through surgical removal of the ovary and uterus [25,26].

Regarding contraceptive delivery methods, oral routes (7/19 studies) and intramuscular (7/19 studies) are the most convenient in wildlife because they are delivered remotely. The remote administration of contraception is considered appropriate for wildlife in urban parks, islands, and enclosed wildlife game reserves [70,71,72,73]. In contrast, in wildlife fertility control, remote delivery or the oral bait delivery of contraceptive agents or any drug is usually accompanied by the risk of consumption by non-target species [74]. These can be a setback to oral contraceptive drug administration. Notwithstanding, oral bait delivery can cover larger free-ranging populations at a lower cost [74].

Furthermore, unlike restraining methods, remote contraceptive administration cannot achieve total dose consumption [65]. In addition, contraceptives that are remotely delivered must be repeated at least once a year because no contraceptive agent that can be delivered remotely seems effective for more than one year [67]. Other methods of administration that necessitate animal restraint, such as subcutaneous (5/19 studies), intravenous (1/19 studies), and invasive surgical procedures (2/19 studies), have been reported to have a negative impact on the welfare of treated animals in addition to the physiological and behavioral effects of the contraceptive agent and or procedures [75]. 

Economic constraints are an additional factor of equal importance in contraception for wildlife. The contraceptive and the labor required to administer it incur costs, which can be substantial [76]. According to Bomford and O’Brien [67], the chosen methods and administration routes should maximize the benefits-to-cost ratio. In addition to providing biological benefits, contraceptives should be economically feasible for implementation, including the cost of developing, registering, and delivering the contraceptive to the target population [74]. 

This analysis acknowledges the differences between captive and free-ranging cynomolgus macaques as a further limitation. Only 2 of the 19 non-hormonal methods (2/19 studies) included in this analysis investigated contraception in free-ranging female cynomolgus macaques, making it hard to determine the actual efficacy of contraception under field conditions. Wildlife contraceptive studies should examine the contraceptive’s effect on sexual behavior, reproductive status, health, and social group interaction of the treated animals. Such longitudinal observation is needed to assess the short- and long-term contraceptive effects [76].

The next obstacle would be to select either permanent or reversible contraception. Permanent contraceptives are better for animal welfare because they are administered less often. One-dose permanent contraception is cheaper and easier to administer. Long-term contraceptives should be used for long-lived animals instead of culling [67]. Reversible contraception also helps preserve the gene pool and control the target population, especially for protected species [77]. Population modeling can be used to decide whether a particular group should use permanent or reversible birth control [78].

## 4. Study Limitation

The need for more focus on contraceptive safety is one of this study’s many flaws. In the future, scientists should study the side effects of contraceptive methods on both male and female cynomolgus macaques and the effectiveness of hormonal contraceptive methods on male cynomolgus macaques. This study also did not examine macaque social behavior concerning contraceptives. Changes in the cynomolgus macaque’s social or sexual behavior could affect the bonds between group members, resulting in an unbalanced social structure. Finally, this analysis only includes a few studies on free-ranging cynomolgus macaques; thus, it may not be possible to draw firm conclusions on the effectiveness of contraception in reducing macaque populations or its effect on human–macaque conflict.

## 5. Conclusions and Recommendations

Despite the challenges, we conclude that hormonal contraception in female long-tailed macaques and non-hormonal contraception in male long-tailed macaques exhibit certain levels of safety and efficacy. The effectiveness of the contraceptive, the administration route, economic feasibility, the distinction between captive and free-ranging cynomolgus macaques, the choice of permanent or reversible contraception, and the literature gap in this field are among the challenges. Nevertheless, there is a lack of research examining the effectiveness of long-term fertility control programs for cynomolgus macaques in addressing the issue of human–macaque conflict. Therefore, future studies on long-tailed macaque contraception should further emphasize solving these challenges to establish that such methods are suitable alternative methods to lethal population control.

## Data Availability

The corresponding authors can provide all information upon request.
